# Naltrexone Inhibits IL-6 and TNFα Production in Human Immune Cell Subsets following Stimulation with Ligands for Intracellular Toll-Like Receptors

**DOI:** 10.3389/fimmu.2017.00809

**Published:** 2017-07-11

**Authors:** Rachel Cant, Angus G. Dalgleish, Rachel L. Allen

**Affiliations:** ^1^Institute for Infection and Immunity, St George’s University of London, London, United Kingdom

**Keywords:** toll-like receptor, naltrexone, interleukin-6, tumor necrosis factor alpha, plasmacytoid dendritic cells, B cells, monocytes

## Abstract

The opioid antagonist naltrexone hydrochloride has been suggested to be a potential therapy at low dosage for multiple inflammatory conditions and cancers. Little is known about the immune-modulating effects of naltrexone, but an effect on the activity of toll-like receptor 4 (TLR4) has been reported. We analyzed the effects of naltrexone hydrochloride on IL-6 secretion by peripheral blood mononuclear cells (PBMC) *in vitro* following stimulation with ligands for TLR4 and for the intracellular receptors TLR7, TLR8, and TLR9. Naltrexone did not affect cell viability or induce apoptosis of PBMC. Intracellular staining demonstrated that naltrexone inhibited production of IL-6 and TNFα by monocyte and plasmacytoid dendritic cell subsets within the PBMC population following treatment with ligands for TLR7/8 and TLR9, respectively. No effect of cytokine production by PBMC following stimulation of TLR4 was observed. Additionally, naltrexone inhibited IL-6 production in isolated monocytes and B cells after TLR7/8 and TLR9 stimulation, respectively, but no effect on IL-6 production in isolated monocytes after TLR4 stimulation was observed. These findings indicate that naltrexone has the potential to modulate the secretion of inflammatory cytokines in response to intracellular TLR activity, supporting the hypothesis that it may have potential for use as an immunomodulator.

## Introduction

Naltrexone hydrochloride is an opioid antagonist used commonly in the treatment of opioid and alcohol dependence (1, 2). Naltrexone specifically inhibits the mu and, to a lesser extent, the delta opioid receptors (3), thus preventing the euphoric effects of alcohol or opioid. It has been suggested that treatment with low-dose naltrexone (LDN) may be beneficial for a range of inflammatory conditions, including Crohn’s disease (4), multiple sclerosis (5), and fibromyalgia (6–8). Reports also describe therapeutic effects of LDN in treatment for cancers including B cell lymphoma (9) and pancreatic cancer (10, 11). The molecular targets and potential immunomodulatory mechanism(s) of action for naltrexone in inflammatory conditions, however, require further investigation. Studies by Zagon et al. indicate that naltrexone can inhibit the non-canonical opioid growth factor receptor, resulting in a decrease in cell proliferation (12–14). Naltrexone and the related opioid antagonist naloxone have also been shown to inhibit the activity of a member of the toll-like receptor (TLR) family, TLR4, in an *in vitro* signaling assay and to reverse neuropathic pain in an animal model (15, 16).

Toll-like receptors recognize conserved molecular patterns and nucleic acids as part of the innate immune response (17). Ten members of the TLR family have been described in humans and these vary in their cellular location; TLR1, TLR2, TLR4, TLR5, TLR6 and TLR10 are expressed on the cell surface, where they can detect components of extracellular pathogens and some self-ligands, while TLR3, TLR7, TLR8, and TLR9 are located within endosomes where they respond to the presence of viral, bacterial, and self-nucleic acids (18). TLRs also vary in their expression profile between immune cell subsets. For example, B cells express TLR1, TLR6, TLR7, and TLR9 (19), while monocytes express TLR1, TLR2, TLR4, TLR7, and TLR8 (19, 20) and plasmacytoid dendritic cells express TLR7 and TLR9 (19, 21).

Although TLRs play a key role in the initiation of immune responses to infection, inappropriate TLR activity and/or recognition of self-ligands are associated with inflammatory conditions and autoimmunity (22). For example, increased expression of TLRs has been observed in peripheral B cells from patients with inflammatory bowel disease (23), while recognition of self-DNA complexes by TLR9 mediates pDC activation in psoriasis (24). TLRs have also been implicated in the tumor microenvironment, with TLR activation linked to angiogenesis, tumor proliferation, and immune evasion (25). Furthermore, some TLR polymorphisms may be associated with development of inflammatory conditions such as Crohn’s disease (26, 27). TLRs have, therefore, been investigated as potential therapeutic targets in patients with these diseases (28, 29).

In this study, we sought to investigate the ability of naltrexone hydrochloride to inhibit the effects of TLR4 signaling in an immune context and to determine whether its inhibitory effects extend to other members of the TLR family. Our results indicate that naltrexone can inhibit production of the inflammatory cytokines IL-6 and TNFα by peripheral blood mononuclear cells (PBMC) following stimulation with known ligands for TLR7, TLR8, and TLR9 but not following stimulation with a TLR4 ligand. Although the interleukin 1 receptor (IL-1R) shares the MyD88 signaling pathway with members of the TLR family, IL-6 secretion following IL-1R stimulation was not affected by naltrexone. Our findings also indicate that naltrexone does not affect cell viability or induce apoptosis within the PBMC population.

## Materials and Methods

### Ethics Statement

This study was carried out in accordance with the recommendations of St George’s, University of London Research Ethics Committee (Protocol Approval SGREC15.0006). All subjects gave written informed consent.

### Cell Culture

Peripheral blood mononuclear cells were isolated from leukocyte cones (NHS Blood Donor Service) by density centrifugation over Histopaque (Sigma-Aldrich) according to the manufacturer’s instructions. PBMC were resuspended at a concentration of 10^6^ PBMC/ml in RPMI-1640 (Sigma-Aldrich) supplemented with 10% fetal bovine serum (Sigma-Aldrich), penicillin, and streptomycin (Sigma-Aldrich). PBMC viability was assessed using trypan blue dye exclusion using the BioRad TC20 Automated Cell Counter (BioRad). PMBC with a viability of above 90% were used in assays. PBMC were plated onto 24 well plates and cultured at standard cell culture conditions at 37°C, 5% CO_2._

### Cell Stimulation

The following ligands were used to stimulate cells: 1 ng/ml LPS-EB Ultrapure, 1µM CPG-ODN 2395, 1µM R848, and 100 ng/ml IL-1 (all Invivogen). For isolated B cell experiments, CD40R was cross-linked using 3 µg/ml CD40-L (R&D Biosource) with anti-HA monoclonal antibody (Sigma-Aldrich) and 20 ng/ml IL-4 (R&D Biosource). Lyophilized ligands were resuspended in endotoxin-free water as detailed in the manufacturer’s instructions. Ligands were further diluted in RPMI before being added to PBMC at the concentrations stated. Naltrexone hydrochloride (Sigma-Aldrich) was resuspended in endotoxin-free water and diluted in RPMI before being added to PBMC at the working concentrations specified.

### Isolation of CD14+ and CD19+ Cells

Positive selection of CD14+ and CD19+ was performed by incubating PBMC with MACS CD14+ and CD19+ microbeads in MACS buffer, according to the manufacturer’s instructions (Miltenyi Biotec). After incubation of cells and microbeads, cells were washed with MACS buffer, resuspended in MACS buffer, and loaded onto a MACS column attached to a magnetic field of a MACS separator. After being washed with MACS buffer three times, the column was removed from the magnetic field and the CD14+ and CD19+ cells were eluted using MACS buffer (Miltenyi Biotec). Purity of above 90% was confirmed by flow cytometry using CD14-VioBlue mIgG1 antibody and CD20 FITC mIgG1 antibody (Miltenyi Biotec).

### IL-6 ELISA

10^6^ PBMC were stimulated with ligands and naltrexone as stated above for 24 h before cell-free supernatants were collected and IL-6 ELISA was performed using an IL-6 ELISA kit (BD Bioscience) as per manufacturer’s instructions. Optical densities were measured using GloMax-Multi+ Microplate with Instinct microplate reader (Promega). Data were then analyzed using a 5-parameter sigmoidal curve on Graph Pad Prism Version 7.

### Intracellular Cytokine Staining

10^6^ PBMC were stimulated with TLR ligand (TLR-L) and naltrexone for 6 h in the presence of brefeldin A (eBioscience) for 4 of those hours. After 6 h, PBMC were washed with PBS and cell surface markers were stained using fluorochrome-conjugated monoclonal antibodies. Antibodies used were CD14-VioBlue, mIgG1, clone TUK4, CD1c-VioBright FITC, mIgG2a clone AD5-8E7, CD303 PE-Vio770, mIgG1, clone AC144 (all Milenyi Biotec) and CD19-PE, mIgG1, clone HIB19 (eBioscience), or appropriate isotype. After washing in PBS, PBMC were fixed and permeabilized using BD cell fixation/permeabilization kit. PBMC were then washed in BD perm/wash buffer and stained for IL-6 and TNF-α using TNFα, hIgG1, clone cA2 (Milentyi Biotec) or IL-6 APC, rIgG1, clone MQ2-13A5 (eBioscience), or appropriate isotype. After washing with BD perm/wash buffer, PBMC were ran on the BD Canto running BD FACSDiva software and analyzed using FlowJo software.

### Flow Cytometry Analysis

Unstained PBMC and fluorescence minus one (FMO) controls, in combination with appropriate isotype controls, were used to determine gating. Figure S4 in Supplementary Material shows the gating strategy, and all flow cytometry data were analyzed using FlowJo software. PBMC population was gated based on the size (FSC) and granularity (SSC) of the cells. CD14+ and CD19+ were used to identify monocytes and B cells, respectively. Within the CD14− CD19− population, myeloid dendritic cells and plasmacytoid dendritic cells were identified by CD1c and CD303 positivity, respectively. To determine the expression of the intracellular cytokines, histograms were generated to determine the percentage of subsets that is positive for the marker or cytokine of interest. IL-6 and TNFα positive and negative populations were gated based on FMO in combination with isotype control. Mean fluorescence intensity of TNFα and IL-6 was also determined.

### Cell Viability

One million PBMC were stimulated with TLR-L and naltrexone for 24 h before being resuspended in 1× annexin V binding buffer (eBioscience) and incubated with 5 µl annexin V APC (eBioscience) for 20 min. Cells were then washed in 1 ml 1× annexin V binding buffer and resuspended in 200 µl 1× annexin V binding buffer. 5 µl 7AAD was then added, and data were collected using the BD Canto. Data were analyzed using FlowJo software.

### Statistics

Data are presented as mean with the SEM, and statistical analysis was performed using GraphPad Prism version 6.07 for Windows. Data were analyzed using an one-way ANOVA and Tukey’s multiple comparison test. A *p* value of below 0.05 was deemed to be significant.

## Results

### Naltrexone Inhibits IL-6 Production Induced after TLR7/8 and TLR9 but Not TLR4 or IL-1R Stimulation

**Figure 1:**
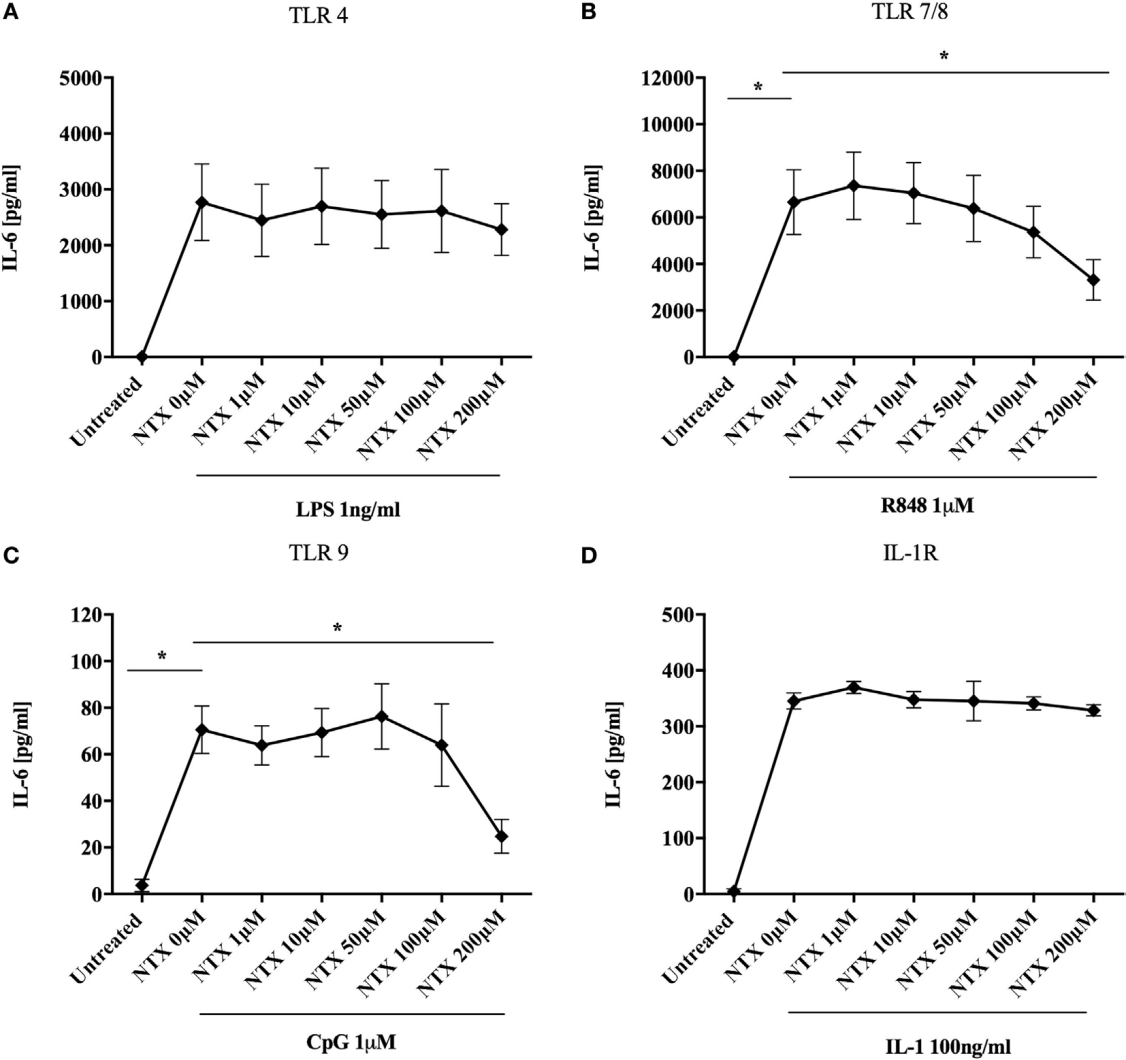
Naltrexone inhibits IL-6 production after toll-like receptor (TLR)7/8 and TLR9 stimulation but not after TLR4 or IL-1 stimulation. 1 × 10^6^ peripheral blood mononuclear cells were incubated with **(A)** 1 ng/ml LPS (TLR4-L), **(B)** 1µM R848 (TLR7/8-L), **(C)** 1µM CpG (TLR9-L), **(D)** 100 ng/ml IL-1 (IL-1R) in the presence or absence of 1–200µM naltrexone for 24 h. Cell-free supernatants were collected and analyzed for IL-6 by ELISA. Data show the mean; SD values are shown and were analyzed using an one-way ANOVA and Tukey’s multiple comparison test (*n* = 5 TLR ligand experiments and *n* = 3 IL-1). *p < 0.05.

It has previously been shown that naltrexone inhibits TLR4 activity both in an *in vitro* assay system and in microglial cells (15, 16). We therefore sought to determine the effect of naltrexone on this and other members of the TLR family in an immune context, focusing on production of IL-6, a key cytokine produced following TLR stimulation. Titrations were performed in order to determine the optimum concentration of TLR-Ls that induce statistically significant IL-6 production in PBMC (Figure S1 in Supplementary Material). PBMC were stimulated with TLR-Ls for TLR4 (LPS 1 ng/ml), TLR7/8 (R848 1µM), and TLR9 (CpG 1µM) in the presence or absence of naltrexone (1–200µM), and IL-6 production was determined by ELISA. Naltrexone had no effect on IL-6 production following TLR4 stimulation (Figure 1A); however, 200µM naltrexone inhibited IL-6 production following stimulation with ligands for TLR7/8 (Figure 1B, *p* < 0.05) and TLR9 (Figure 1C, *p* < 0.05) (these data are also presented as dose response curves in Figure S2 in Supplementary Material). As R848 is a ligand for both TLR7 and TLR8, we sought to determine if naltrexone inhibits IL-6 production after TLR7 (R837 3 µg/ml) or TLR8 (ssRNA 0.5 µg/ml) stimulation. Naltrexone inhibited IL-6 production after both TLR7 and TLR8 stimulation in a dose-dependent manner, although this did not reach significance (Figure S3 in Supplementary Material). As TLR7, TLR8, and TLR9 signal *via* the MyD88 pathway, whereas TLR4 can signal *via* both MyD88-dependent and -independent pathways (28, 30), we hypothesized that naltrexone may affect the MyD88-dependent signaling pathway and that any effects of naltrexone on IL-6 secretion *via* TLR4 were compensated for by signaling through the MyD88-independent pathway. Stimulation of the IL-1R also results in induction of the MyD88-dependent pathway and the secretion of IL-6. However, when PBMC were stimulated with IL-1 (100 ng/ml) in the presence of naltrexone (1–200µM), no effect on IL-6 production observed (Figure 1D).

### Naltrexone Inhibits Intracellular Cytokine Production after TLR7/8 and TLR9 Stimulation but Not TLR4 Stimulation

**Figure 2:**
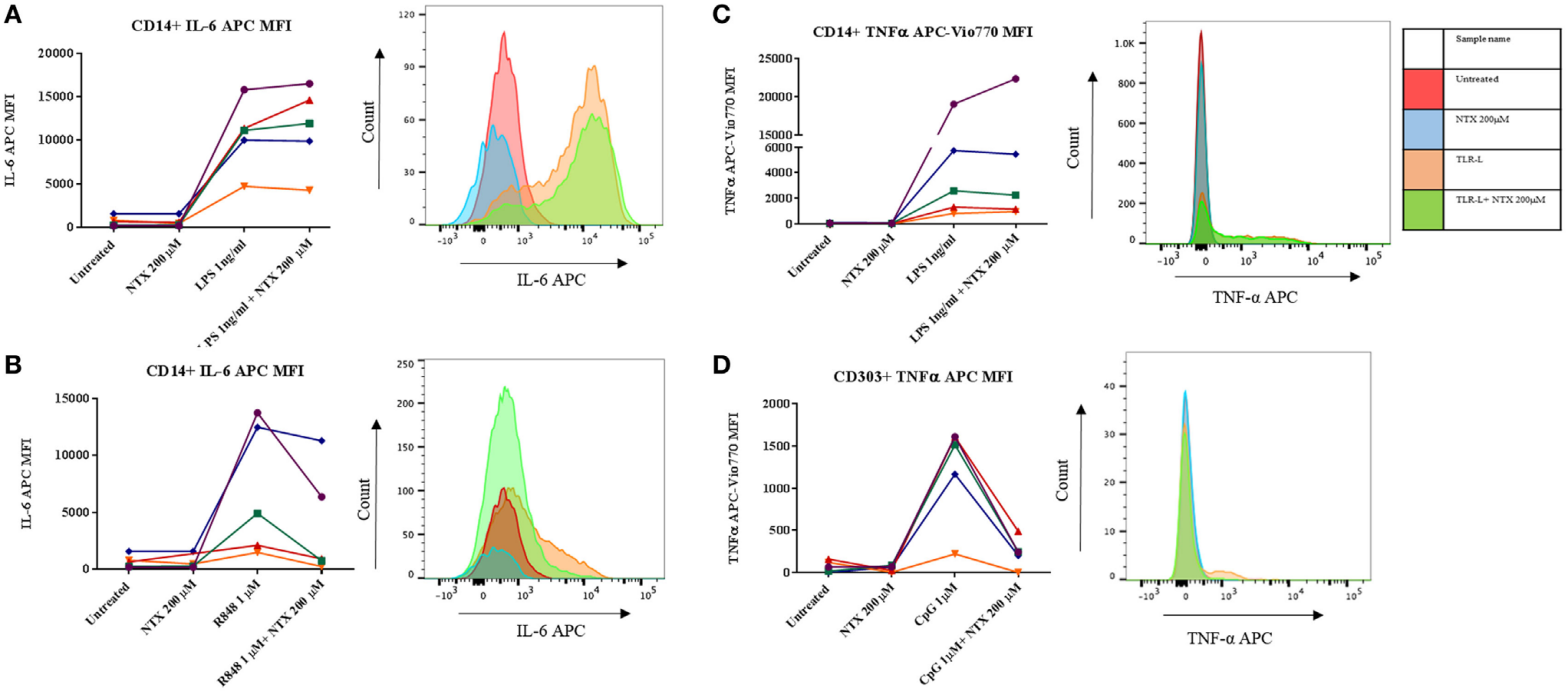
Intracellular cytokine staining for TNFα and IL-6 in monocytes and plasmacytoid dendritic cells. 1 × 10^6^ peripheral blood mononuclear cells (PBMC) were incubated with either LPS 1 ng/ml **(A,C)**, R848 1µM **(B)**, or CpG 1µM **(D)** and 200 µM naltrexone for 6 h in the presence of brefeldin A for 4 of those hours. After 6 h, PBMC were stained using antibody panel shown in Figure S4 in Supplementary Material and stained for either intracellular IL-6 or TNF-α. Results show the mean fluorescence intensity (MFI) of IL-6 or TNF-α within that subset from 5 donors. Histograms are representative of 5 independent experiments.

In order to determine which subset(s) of cells within the PBMC population were effected by naltrexone, intracellular cytokine staining was performed. In addition to IL-6 production we also examined the effect naltrexone has on another signature cytokine produced after TLR stimulation, TNF-α. PBMC were stimulated with TLR-L (LPS 1 ng/ml, R848 1µM, and CpG 1µM) and 200µM naltrexone for 6 h, with the addition of brefeldin A after 2 h. PBMC were then stained for cell surface markers, as shown in Figure S4 in Supplementary Material, to identify monocytes (CD14+), B cells (CD19+), myeloid dendritic cells (CD14− CD19− CD1c+, mDCs), and plasmacytoid dendritic cells (CD14− CD19− CD1c− CD303+, pDCs) and for intracellular IL-6 or TNFα (Figure 2). Monocytes were identified as a major source of IL-6 following LPS and R848 stimulation (Figures 2A,B). In line with our observations from ELISA data described above, naltrexone did not appear to affect IL-6 production by CD14+ cells following LPS stimulation (Figure 2B). A decrease in IL-6 production in monocytes after R848 and naltrexone incubation was observed, although this did not reach statistical significance (Figure 2B). Incubation with the TLR9 ligand CpG induced IL-6 production in B cells however, this was not altered by the addition of 200μM naltrexone to cultures (data not shown). Furthermore, at the time point examined, no cytokine production was observed in mDC following incubation with LPS, R848, or CpG (data not shown). TNFα was induced following LPS and CpG stimulation in monocytes and pDCs, respectively (Figures 2C,D). Similar to the results observed for IL-6, naltrexone did not affect TNF-α production following LPS stimulation in monocytes (Figure 2C), whereas naltrexone did inhibit TNF-α production in plasmacytoid dendritic cells following TLR9 stimulation (Figure 2D, *p* < 0.05).

### Naltrexone Inhibits IL-6 Production in Isolated Monocytes and B Cells after TLR7/8 and TLR9 Stimulation, Respectively, but Has No Effect on IL-6 Production in Isolated Monocytes after TLR4 Stimulation

**Figure 3:**
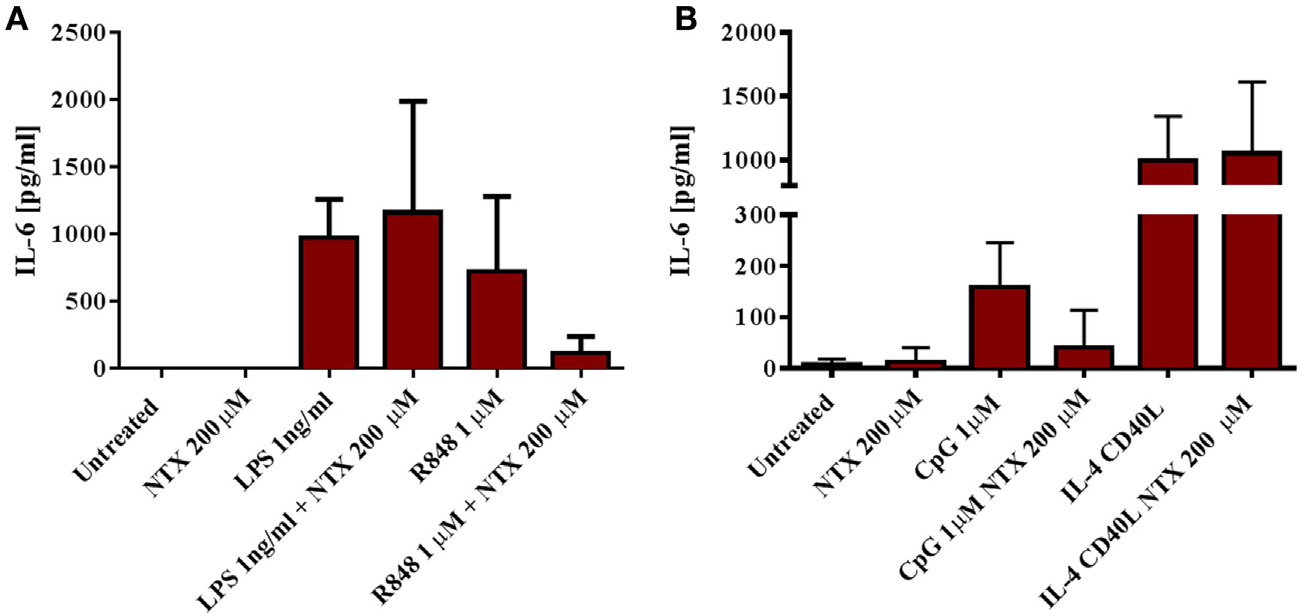
Naltrexone inhibits IL-6 production in isolated monocytes and B cells after toll-like receptor (TLR)7/8 and TLR9 stimulation, respectively, but has no effect on IL-6 production in isolated monocytes after TLR4 stimulation. **(A)** CD14+ monocytes were isolated from peripheral blood mononuclear cells (PBMC) using magnetic bead isolation. 1 × 10^5^ CD14+ cells were incubated with 1 ng/ml LPS (TLR4-L) or 1µM R848 (TLR7/8-L), in the presence or absence of 200µM naltrexone for 24 h. Cell-free supernatants were collected and analyzed for IL-6 by ELISA. **(B)** CD19+ B cells were isolated from PBMC using magnetic bead isolation. 10^5^ B cells were incubated with 1µM CpG or 3 µg/ml CD40-L and 20 ng/ml IL-4, with or without 200µM naltrexone for 24 h. IL-6 production was measured in cell-free supernatants by ELISA. Data show the mean and SD values (*n* = 4).

To further confirm that naltrexone does not inhibit cytokine production after TLR4 stimulation, we isolated CD14+ monocytes from PBMC using magnetic bead isolation. Isolated CD14 cells were then stimulated with LPS 1 ng/ml and R848 1µM in the presence or absence of naltrexone 200µM for 24 h. Cell-free supernatants were analyzed for the presence of IL-6 by ELISA. Similar to the data obtained from intracellular cytokine analysis described above, naltrexone inhibited IL-6 production in monocytes following R848 stimulation, but no effect on LPS-induced IL-6 production was observed (Figure 3A). Additionally, within the PBMC population, TLR9 is predominately expressed on B cells. Therefore, to determine if naltrexone effects IL-6 production in isolated B cells after TLR9 stimulation, B cells were stimulated with CpG 1µM in the presence of 200µM naltrexone for 24 h. Naltrexone inhibited IL-6 production after TLR9 stimulation but not after cross-linking of CD40R and stimulation with IL-4 (Figure 3B).

### Naltrexone Does Not Affect PBMC Viability

**Figure 4:**
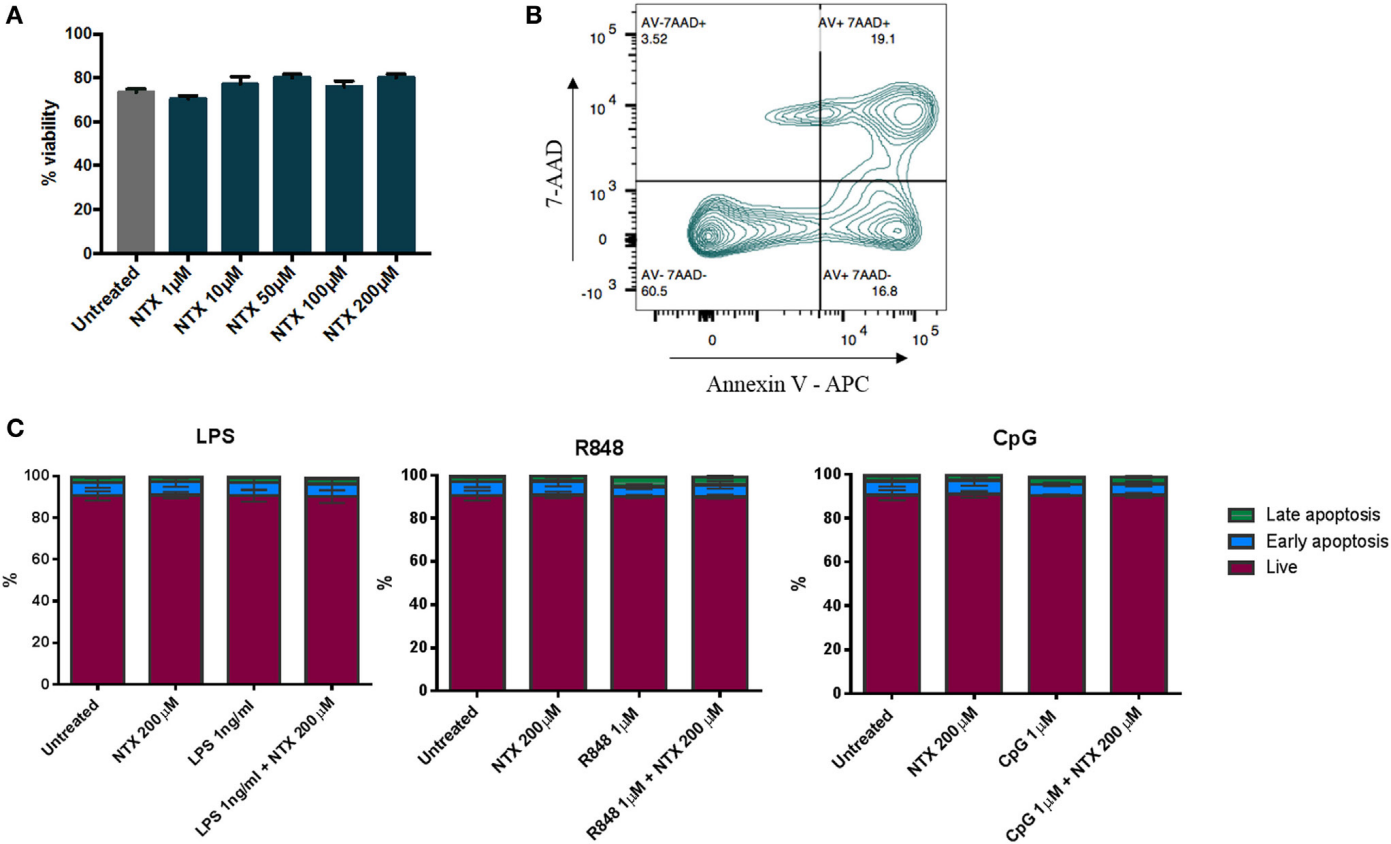
Toll-like receptor ligand (TLR-L) and naltrexone does not affect the viability of peripheral blood mononuclear cells (PBMC). **(A)** 1 × 10^6^ PBMC were incubated with 1–200µM naltrexone for 24 h before percentage viability was assessed using trypan blue exclusion. **(B,C)** 1 × 10^6^ PBMC were incubated with 1 ng/ml LPS (TLR4-L), 1µM R848 (TLR7/8-L), 1µM CpG (TLR9-L), and 200µM naltrexone for 24 h. PBMC were incubated with annexin V and 7AAD before being analyzed by flow cytometry. Figure 4B shows the gating strategy, and Figure 4C shows results from 4 donors. AV−7AAD− are viable cells, AV+7AAD− are in early apoptosis, and AV+7AAD+ are in late apoptosis.

To ensure that the decreases in IL-6 production we observed in the presence of naltrexone were not due to a loss of cell numbers, viability was assessed by trypan blue staining following PBMC incubation with naltrexone (1–200µM) for 24 h. No change in cell viability was observed (Figure 4A). Additionally, to determine if naltrexone induces apoptosis, annexin V and 7AAD staining was performed on PBMC following 24 h incubation with naltrexone and TLR-Ls (Figure 4B). As shown in Figure 4C, there was no evidence to suggest that TLR-Ls or naltrexone incubation induce apoptosis in PBMC at the concentrations tested in this study.

## Discussion

Through their roles as mediators of both innate and adaptive immune functions, TLRs are powerful agents within the immune system. Intracellular TLRs have been investigated as potential therapeutic targets for the treatment of inflammatory diseases and cancer (29, 31–33). Inhibition of TLR-mediated functions by naltrexone could, therefore, indicate a potential immunomodulatory relevance for this drug in the treatment of inflammatory disease. In this study, we show that naltrexone can inhibit the production of cytokines by PBMC following treatment with ligands for the intracellular receptors TLR7, TLR8, and TLR9. Flow cytometric analysis of individual cell subsets indicated that naltrexone inhibited IL-6 production by monocytes in response to TLR7/8 ligands and TNFα production by pDCs in response to TLR9 ligand. These reductions in cytokine secretion did not appear to result from a loss of cell viability, as no significant effects on cell numbers or expression of apoptotic markers was observed.

One unexpected finding of this study was that naltrexone did not inhibit cytokine secretion by immune cells following stimulation with LPS, a ligand for TLR4. Previously published work had shown that naltrexone and naloxone can inhibit TLR4-dependent microglial activation, neurodegeneration, and nitric oxide production (16, 34) and have identified the LPS binding site of the TLR4 co-receptor MD2 as a binding site for the drug (35, 36). Previous studies documented the effect of the purified isomers of naltrexone on TLR4, whereas our study used naltrexone-HCl, a hydrochloride salt commonly prescribed in tablet form to patients. Both isomers have shown to bind MD2 and inhibit TLR4 activity (34, 35) in a HEK-293 reporter cell line and rat microglial cells. The (+)-isomer of naltrexone does not act on opioid receptors, which may be beneficial for use in therapies directed at alternative receptors. Further investigations will be necessary to determine the effects of different naltrexone isomers on TLR7, TLR8, and TLR9, which are intracellular and do not associate with MD2.

Our experiments have shown that naltrexone can inhibit cytokine secretion in response to TLR ligands, although further work will be required to determine the mechanism(s) of action involved. Each of the TLR investigated in the current study (TLR4, TLR7, TLR8, and TLR9) signal through the MyD88-dependent pathway, although TLR4 can also signal *via* the MyD88-independent TRIF pathway. It could be hypothesized that inhibition of cytokine production following TLR7, TLR8, or TLR9 stimulation results from inhibition of the MyD88 pathway, and that the observed lack of TLR4 antagonism in our experiments results from signaling *via* TRIF pathway, which can induce delayed NFκB activation and resultant IL-6 and TNFα production. However, previously published work has suggested that naltrexone inhibits phosphorylation of IRF3, a transcription factor that downstream of TRIF activation (34). Also, our observation that naltrexone did not inhibit cytokine secretion in response to stimulation of the IL-1 receptor, which also signals by the MyD88 pathway, would support an interaction upstream of this adaptor protein. Further investigations are required to determine the signaling pathways regulated by naltrexone and how this can account for TLRs effected. Furthermore, intracellular cytokine assays in this study examined the effect of naltrexone on the production of IL-6 and TNFα after 6-h incubation. This approach does not provide information of the potential effect of naltrexone on cytokine kinetics. More detailed analyses determining the effect of naltrexone on cytokine production at different time points would be required in order to investigate whether naltrexone may delay cytokine production.

The reduction of cytokine secretion observed in the presence of naltrexone in our studies did not result from a reduction in cell numbers or a decrease in cell viability, as evidenced by dye exclusion and flow cytometric analysis for markers of apoptosis. This provides further support for our theory that naltrexone can modulate immune cell functions through influencing TLR activity, thus extending the known immune effects of the drug beyond the previously documented inhibition of lymphocyte proliferation *in vitro* and *in vivo* (37, 38). However, this study was only performed within the whole PBMC population, and therefore it is possible that subtle changes in individual immune cell subsets within the PBMC population would not be detected. Future studies would consider the viability of the individual immune subsets after incubation with naltrexone.

An ability to modulate TLR activity would provide justification to support the use of naltrexone for the treatment of inflammatory conditions in which these receptors play a pathogenic role. For example, recognition of self-DNA/protein complexes by TLR9 mediates pDC activation in psoriasis, breaking self-immune tolerance (24). Members of the TLR family, including TLR9, are often ectopically expressed in tumors (39, 40), can induce tumor invasion *in vitro* (41), and may be an indicator of poor prognosis *in vivo*. Similarly, expression of TLR9 has been found to correlate with the invasive and metastatic potential of pancreatic carcinoma (42).

Future studies will be required to investigate whether and how naltrexone inhibits TLR-mediated inflammatory effects in other cell types such as mucosal epithelial cells (43), and whether exposure to naltrexone results in upregulation of TLR in a similar manner to that seen for its opioid receptor targets (44, 45). Additionally, while this study investigated the effect of naltrexone on IL-6 and TNFα production, further work examining other cytokines, such as IL-12p70, which might be induced after multiple TLR stimulation, would provide further insights into the ability of naltrexone to modulate immune subset activity. It will also be important to consider how the potential pleiotropic effects of naltrexone, including inhibition of TLR-mediated functions, inhibition of cellular proliferation, and other opioid receptor-mediated activity, might contribute to its use in the treatment of inflammatory conditions. In this context, it is important to note that previous studies in inflammatory diseases and cancer have adopted an LDN regime as opposed to the dosages used in the treatment of opioid and alcohol dependency. Nanomolar, but not micromolar, doses of naltrexone were previously seen in studies by Liu et al. to result in upregulation of pro-apoptotic genes, rendering tumor cells more susceptible to chemotherapy (46). It may, therefore, be necessary to identify suitable dosage regimes to obtain optimal therapeutic effects on individual target pathways in different diseases.

## Ethics Statement

This study was carried out in accordance with the recommendations of St George’s, University of London Research Ethics Committee (Protocol Approval SGREC15.0006). All subjects gave written informed consent.

## Author Contributions

AD and RA conceived the original idea for the study. RC and RA designed the experiments and prepared the manuscript. RC performed experiments and analyzed the data. All authors read and approved the manuscript.

## Conflict of Interest Statement

RA and AD are listed as inventors on a patent that describes the use of naltrexone as a TLR9 antagonist, which has been assigned to the Institute for Cancer Vaccines and Immunotherapy. RC declares no competing financial interests.

## References

[1] Krystal JH Cramer JA Krol WF Kirk GF Rosenheck RA. Naltrexone in the treatment of alcohol dependence. N Engl J Med (2001) 345:1734–9. 10.1056/NEJMoa011127 11742047

[2] Lee JD Friedmann PD Kinlock TW Nunes EV Boney TY Hoskinson RA Jr Extended-release naltrexone to prevent opioid relapse in criminal justice offenders. N Engl J Med (2016) 374:1232–42. 10.1056/NEJMoa1505409 27028913 PMC5454800

[3] Weerts EM Kim YK Wand GS Dannals RF Lee JS Frost JJ Differences in δ- and µ-opioid receptor blockade measured by positron emission tomography in naltrexone-treated recently abstinent alcohol-dependent subjects. Neuropsychopharmacology (2008) 33:653–65. 10.1038/sj.npp.1301440 17487229

[4] Smith JP Bingaman SI Ruggiero F Mauger DT Mukherjee A McGovern CO Therapy with the opioid antagonist naltrexone promotes mucosal healing in active Crohn’s disease: a randomized placebo-controlled trial. Dig Dis Sci (2011) 56:2088–97. 10.1007/s10620-011-1653-7 21380937 PMC3381945

[5] Cree BAC Kornyeyeva E Goodin DS. Pilot trial of low-dose naltrexone and quality of life in multiple sclerosis. Ann Neurol (2010) 68:145–50. 10.1002/ana.22006 20695007

[6] Younger JW Zautra AJ Cummins ET. Effects of naltrexone on pain sensitivity and mood in fibromyalgia: no evidence for endogenous opioid pathophysiology. PLoS One (2009) 4:e5180. 10.1371/journal.pone.0005180 19365548 PMC2664472

[7] Younger J Mackey S. Fibromyalgia symptoms are reduced by low-dose naltrexone: a pilot study. Pain Med (2009) 10:663–72. 10.1111/j.1526-4637.2009.00613.x 19453963 PMC2891387

[8] Younger J Noor N McCue R Mackey S. Low-dose naltrexone for the treatment of fibromyalgia: findings of a small, randomized, double-blind, placebo-controlled, counterbalanced, crossover trial assessing daily pain levels. Arthritis Rheum (2013) 65:529–38. 10.1002/art.37734 23359310

[9] Berkson BM Rubin DM Berkson AJ Reversal of signs and symptoms of a B-cell lymphoma in a patient using only low-dose naltrexone. Integr Cancer Ther (2007) 6:293–6. 10.1177/1534735407306358 17761642

[10] Berkson BM Rubin DM Berkson AJ. Revisiting the ALA/N (alpha-lipoic acid/low-dose naltrexone) protocol for people with metastatic and nonmetastatic pancreatic cancer: a report of 3 new cases. Integr Cancer Ther (2009) 8:416–22. 10.1177/1534735409352082 20042414

[11] Berkson BM Rubin DM Berkson AJ The long-term survival of a patient with pancreatic cancer with metastases to the liver after treatment with the intravenous α-lipoic acid/low-dose naltrexone protocol. Integr Cancer Ther (2006) 5:83–9. 10.1177/1534735405285901 16484716

[12] Donahue RN McLaughlin PJ Zagon IS. Cell proliferation of human ovarian cancer is regulated by the opioid growth factor-opioid growth factor receptor axis. Am J Physiol Regul Integr Comp Physiol (2009) 296:R1716–25. 10.1152/ajpregu.00075.2009 19297547

[13] Donahue RN McLaughlin PJ Zagon IS. Low-dose naltrexone suppresses ovarian cancer and exhibits enhanced inhibition in combination with cisplatin. Exp Biol Med (Maywood) (2011) 236:883–95. 10.1258/ebm.2011.011096 21685240

[14] Cheng F McLaughlin PJ Verderame MF Zagon IS. The OGF-OGFr axis utilizes the p16INK4a and p21WAF1/CIP1 pathways to restrict normal cell proliferation. Mol Biol Cell (2009) 20:319–27. 10.1091/mbc.E08-07-0681 18923142 PMC2613082

[15] Grace PM Shimizu K Strand KA Rice KC Deng G Watkins LR (+)-Naltrexone is neuroprotective and promotes alternative activation in the mouse hippocampus after cardiac arrest/cardiopulmonary resuscitation. Brain Behav Immun (2015) 48:115–22. 10.1016/j.bbi.2015.03.005 25774010 PMC5548128

[16] Hutchinson MR Zhang Y Brown K Coats BD Shridhar M Sholar PW Non-stereoselective reversal of neuropathic pain by naloxone and naltrexone: involvement of toll-like receptor 4 (TLR4). Eur J Neurosci (2008) 28:20–9. 10.1111/j.1460-9568.2008.06321.x 18662331 PMC2588470

[17] Kawai T Akira S. The role of pattern-recognition receptors in innate immunity: update on toll-like receptors. Nat Immunol (2010) 11:373–84. 10.1038/ni.1863 20404851

[18] Gay NJ Symmons MF Gangloff M Bryant CE. Assembly and localization of toll-like receptor signalling complexes. Nat Rev Immunol (2014) 14:546–58. 10.1038/nri3713 25060580

[19] Hornung V Rothenfusser S Britsch S Krug A Jahrsdörfer B Giese T Quantitative expression of toll-like receptor 1–10 mRNA in cellular subsets of human peripheral blood mononuclear cells and sensitivity to CpG oligodeoxynucleotides. J Immunol (2002) 168:4531–7. 10.4049/jimmunol.168.9.4531 11970999

[20] Visintin A Mazzoni A Spitzer JH Wyllie DH Dower SK Segal DM. Regulation of toll-like receptors in human monocytes and dendritic cells. J Immunol (2001) 166:249–55. 10.4049/jimmunol.166.1.249 11123299

[21] Reizis B Bunin A Ghosh HS Lewis KL Sisirak V. Plasmacytoid dendritic cells: recent progress and open questions. Annu Rev Immunol (2011) 29:163–83. 10.1146/annurev-immunol-031210-101345 21219184 PMC4160806

[22] Fischer M Ehlers M. Toll-like receptors in autoimmunity. Ann N Y Acad Sci (2008) 1143:21–34. 10.1196/annals.1443.012 19076342

[23] Berkowitz D Peri R Lavy A Kessel A. Increased toll-like receptor 9 expression by B cells from inflammatory bowel disease patients. Hum Immunol (2013) 74(12):1519–23. 10.1016/j.humimm.2013.08.285 24007656

[24] Lande R Gregorio J Facchinetti V Chatterjee B Wang YH Homey B Plasmacytoid dendritic cells sense self-DNA coupled with antimicrobial peptide. Nature (2007) 449:564–9. 10.1038/nature06116 17873860

[25] Sato Y Goto Y Narita N Hoon DSB Cancer cells expressing toll-like receptors and the tumor microenvironment. Cancer Microenviron (2009) 2(Suppl 1):205–14. 10.1007/s12307-009-0022-y 19685283 PMC2756339

[26] Tolentino YFM Elia PP Fogaça HS Carneiro AJV Zaltman C Moura-Neto R Common NOD2/CARD15 and TLR4 polymorphisms are associated with Crohn’s disease phenotypes in southeastern Brazilians. Dig Dis Sci (2016) 61:2636–47. 10.1007/s10620-016-4172-8 27107867

[27] Bank S Andersen PS Burisch J Pedersen N Roug S Galsgaard J Polymorphisms in the toll-like receptor and the IL-23/IL-17 pathways were associated with susceptibility to inflammatory bowel disease in a Danish cohort. PLoS One (2015) 10:e0145302. 10.1371/journal.pone.0145302 26698117 PMC4689491

[28] Kanzler H Barrat FJ Hessel EM Coffman RL. Therapeutic targeting of innate immunity with toll-like receptor agonists and antagonists. Nat Med (2007) 13:552–9. 10.1038/nm1589 17479101

[29] Rahmani F Rezaei N. Therapeutic targeting of toll-like receptors: a review of toll-like receptors and their signaling pathways in psoriasis. Expert Rev Clin Immunol (2016) 12(12):1289–98. 10.1080/1744666X.2016.1204232 27359083

[30] O’Neill LAJ. When signaling pathways collide: positive and negative regulation of toll-like receptor signal transduction. Immunity (2008) 29:12–20. 10.1016/j.immuni.2008.06.004 18631453

[31] Jiang W Zhu FG Bhagat L Yu D Tang JX Kandimalla ER A toll-like receptor 7, 8, and 9 antagonist inhibits Th1 and Th17 responses and inflammasome activation in a model of IL-23-induced psoriasis. J Invest Dermatol (2013) 133:1777–84. 10.1038/jid.2013.57 23370539

[32] Mohamed FE Al-Jehani RM Minogue SS Andreola F Winstanley A Olde Damink SW Effect of toll-like receptor 7 and 9 targeted therapy to prevent the development of hepatocellular carcinoma. Liver Int (2015) 35:1063–76. 10.1111/liv.12626 24990399

[33] Junt T Barchet W. Translating nucleic acid-sensing pathways into therapies. Nat Rev Immunol (2015) 15:529–44. 10.1038/nri3875 26292638

[34] Wang X Zhang Y Peng Y Hutchinson MR Rice KC Yin H Pharmacological characterization of the opioid inactive isomers (+)-naltrexone and (+)-naloxone as antagonists of toll-like receptor 4. Br J Pharmacol (2016) 173:856–69. 10.1111/bph.13394 26603732 PMC4761092

[35] Northcutt AL Hutchinson MR Wang X Baratta MV Hiranita T Cochran TA DAT isn’t all that: cocaine reward and reinforcement require toll-like receptor 4 signaling. Mol Psychiatry (2015) 20:1525–37. 10.1038/mp.2014.177 25644383 PMC4523496

[36] Hutchinson MR Northcutt AL Hiranita T Wang X Lewis SS Thomas J Opioid activation of toll-like receptor 4 contributes to drug reinforcement. J Neurosci (2012) 32:11187–200. 10.1523/JNEUROSCI.0684-12.2012 22895704 PMC3454463

[37] McLaughlin PJ McHugh DP Magister MJ Zagon IS. Endogenous opioid inhibition of proliferation of T and B cell subpopulations in response to immunization for experimental autoimmune encephalomyelitis. BMC Immunol (2015) 16:24. 10.1186/s12865-015-0093-0 25906771 PMC4407783

[38] Zagon IS Donahue RN Bonneau RH McLaughlin PJ T lymphocyte proliferation is suppressed by the opioid growth factor ([Met5]-enkephalin)-opioid growth factor receptor axis: implication for the treatment of autoimmune diseases. Immunobiology (2011) 216:579–90. 10.1016/j.imbio.2010.06.001 20965606

[39] Pinto A Morello S Sorrentino R. Lung cancer and toll-like receptors. Cancer Immunol Immunother (2011) 60:1211–20. 10.1007/s00262-011-1057-8 21789594 PMC11029286

[40] Sandholm J Kauppila JH Pressey C Tuomela J Jukkola-Vuorinen A Vaarala M Estrogen receptor-α and sex steroid hormones regulate toll-like receptor-9 expression and invasive function in human breast cancer cells. Breast Cancer Res Treat (2012) 132:411–9. 10.1007/s10549-011-1590-3 21607583

[41] Qiu J Shao S Yang G Shen Z Zhang Y. Association of toll like receptor 9 expression with lymph node metastasis in human breast cancer. Neoplasma (2011) 58:251–5. 10.4149/neo_2011_03_251 21391743

[42] Wu HQ Wang B Zhu SK Tian Y Zhang JH Wu HS. Effects of CPG ODN on biological behavior of PANC-1 and expression of TLR9 in pancreatic cancer. World J Gastroenterol (2011) 17:996–1003. 10.3748/wjg.v17.i8.996 21448350 PMC3057161

[43] Gribar SC Anand RJ Sodhi CP Hackam DJ. The role of epithelial toll-like receptor signaling in the pathogenesis of intestinal inflammation. J Leukoc Biol (2008) 83:493–8. 10.1189/jlb.0607358 18160540

[44] McLaughlin PJ Stucki JK Zagon IS. Modulation of the opioid growth factor ([Met(5)]-enkephalin)-opioid growth factor receptor axis: novel therapies for squamous cell carcinoma of the head and neck. Head Neck (2012) 34:513–9. 10.1002/hed.21759 21584896

[45] Bigliardi PL Stammer H Jost G Rufli T Büchner S Bigliardi-Qi M. Treatment of pruritus with topically applied opiate receptor antagonist. J Am Acad Dermatol (2007) 56:979–88. 10.1016/j.jaad.2007.01.007 17320241

[46] Liu WM Scott KA Dennis JL Kaminska E Levett AJ Dalgleish AG. Naltrexone at low doses upregulates a unique gene expression not seen with normal doses: implications for its use in cancer therapy. Int J Oncol (2016) 49:793–802. 10.3892/ijo.2016.3567 27279602

