# Supplementary figures and images for: Naltrexone Inhibits IL-6 and TNFα Production in Human Immune Cell Subsets following Stimulation with Ligands for Intracellular Toll-Like Receptors
Source: Front Immunol. 2017 Jul 11;8:809. doi: 10.3389/fimmu.2017.00809 (PMC5504148; doi:10.3389/fimmu.2017.00809)

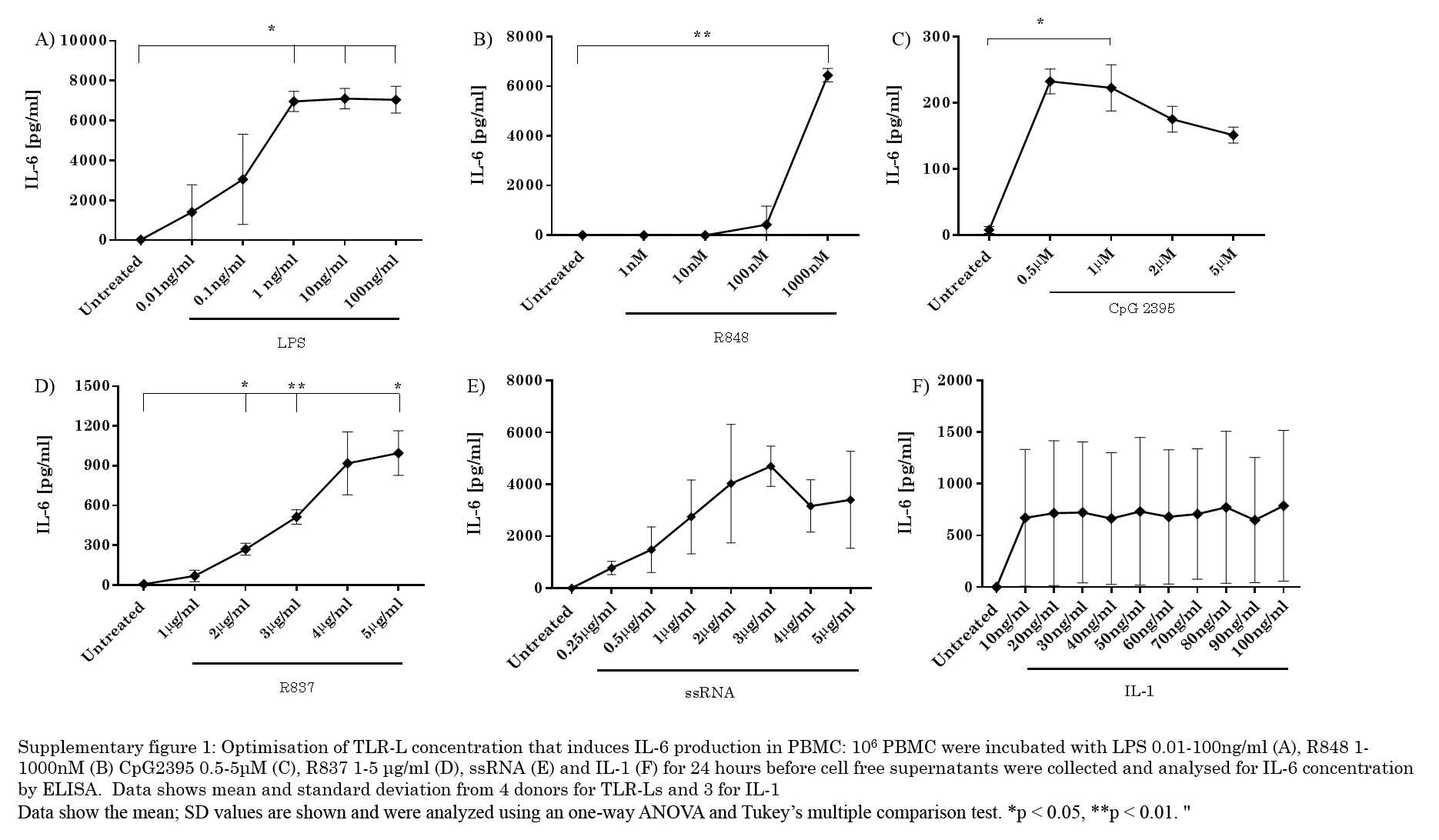

Supplement: Supplementary file 1 [file image_1.tif]

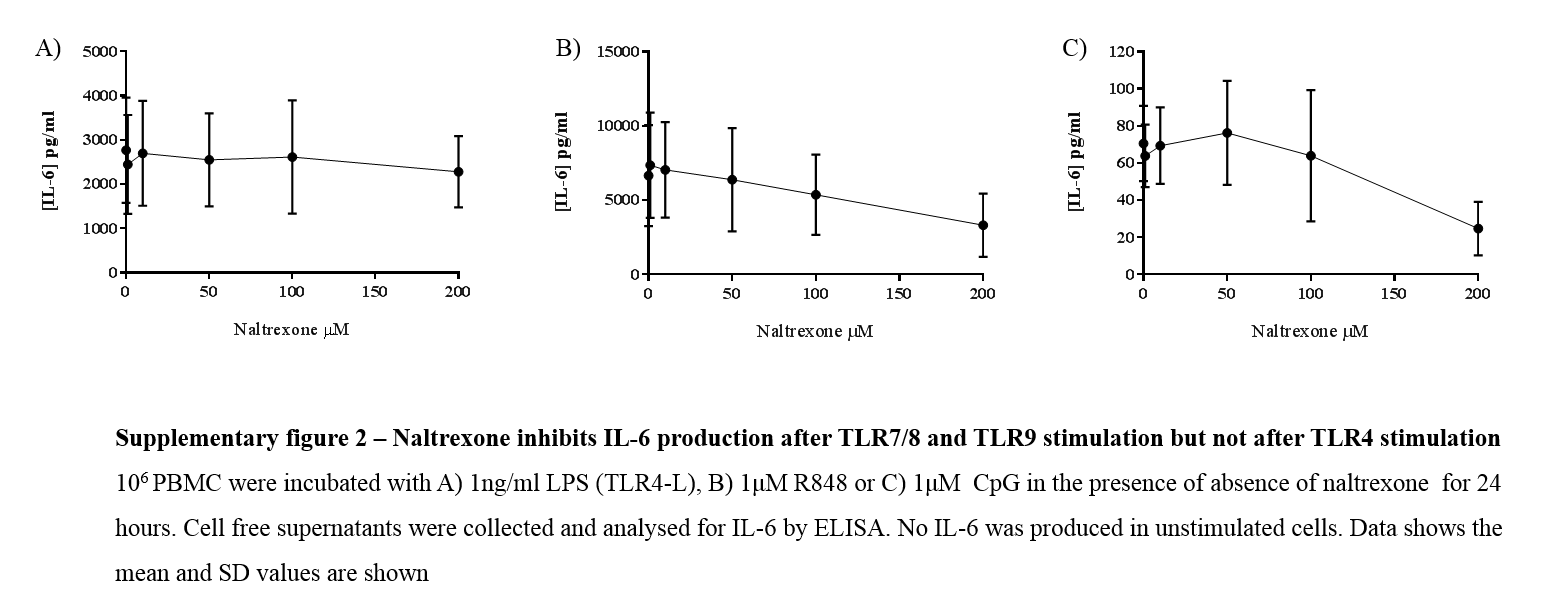

Supplement: Supplementary file 2 [file image_2.tif]

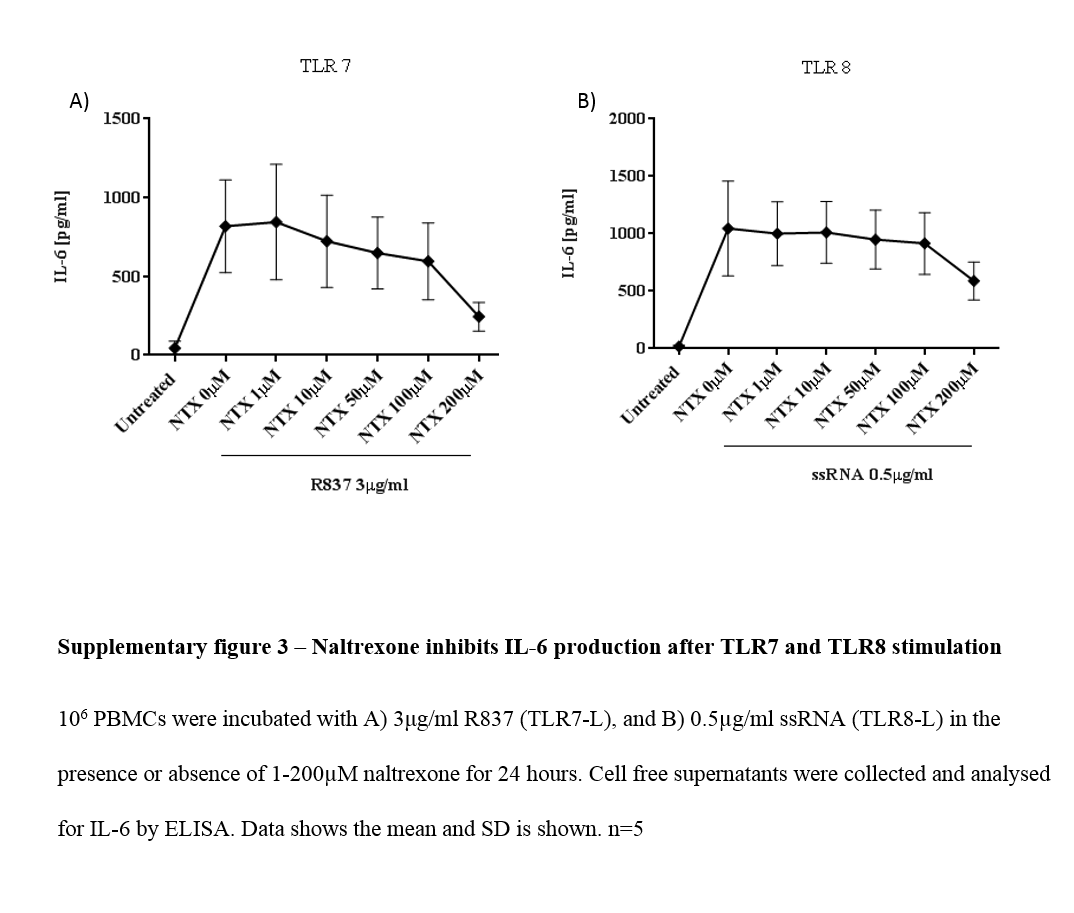

Supplement: Supplementary file 3 [file image_3.tif]

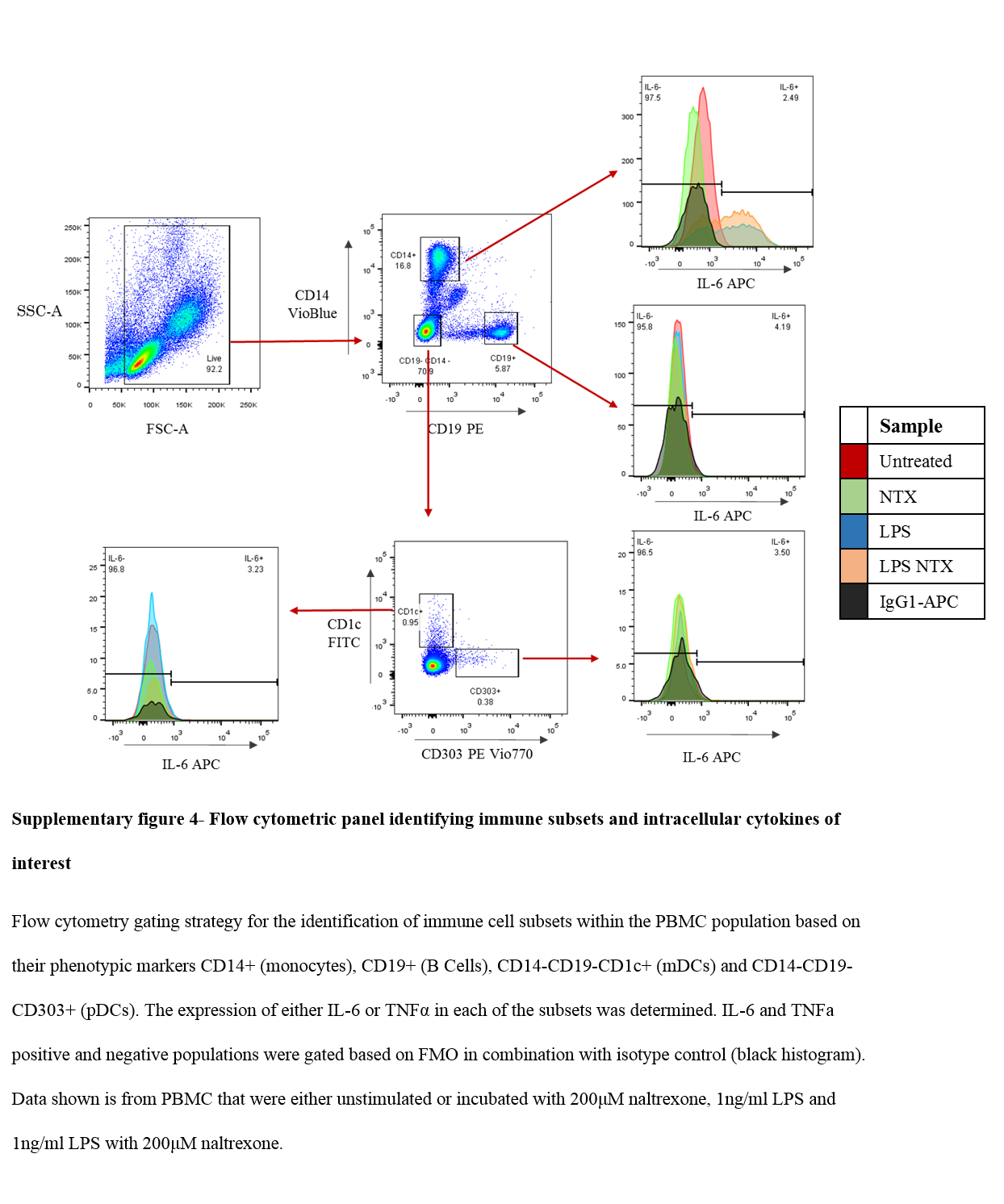

Supplement: Supplementary file 4 [file image_4.tif]
